# The role of community pharmacists and their perception towards antimicrobial stewardship in Baghdad, Iraq

**DOI:** 10.1002/hcs2.92

**Published:** 2024-04-11

**Authors:** Akram Alkadhimi, Omar T. Dawood, Amer H. Khan

**Affiliations:** ^1^ Discipline of Clinical Pharmacy, School of Pharmaceutical Sciences Universiti Sains Malaysia Penang Malaysia; ^2^ College of Pharmacy Ninevah University Mosul Iraq

**Keywords:** antimicrobial stewardship, community pharmacists, perception

## Abstract

**Background:**

This study aimed to assess the role of community pharmacists and their perception toward antimicrobial stewardship, in addition to identifying factors influencing their perception and practices in community pharmacy.

**Methods:**

A cross‐sectional study was carried out among community pharmacists regarding antimicrobial stewardship. Convenience sampling was used to obtain the required sample from a community pharmacy in Baghdad. In total, 381 participants have completed the survey.

**Results:**

The majority of the participants (85.6%) strongly agreed/agreed that “antimicrobial stewardship programs reduce the problems of antibiotic resistance”; and 85.5% of them strongly agreed/agreed that community pharmacists required adequate training on antibiotics use. In addition, high percent of community pharmacists (88.4%) strongly agreed/agreed that pharmacists have a responsibility to take a prominent role in antimicrobial stewardship programs and infection‐control programs in the health system. The total score of perception was significantly influenced by older age groups, postgraduate degrees, and experience of 6–10 years (*p* < 0.001). This study also showed that 65.4% of pharmacists always/often advise patients to continue the full course of antimicrobials, and 64.9% of them reported always/often considering clinical and safety parameters before dispensing antibiotics. The role of pharmacists was significantly influenced by the younger age group, females, higher degree in pharmacy, experience of 3–5 years, and medical complex pharmacy (*p* < 0.001).

**Conclusion:**

Community pharmacists have a good perception toward antimicrobial stewardship programs, but their role is still limited. More efforts are needed to design better strategies for antimicrobial stewardship in community pharmacy.

## Graphical abstract

The role of community pharmacists and their perception toward antimicrobial stewardship in Baghdad, Iraq.

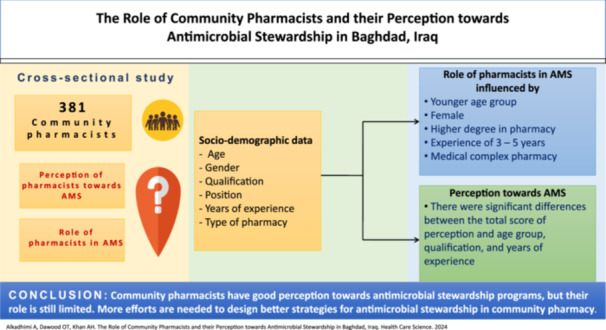

**Abbreviations**
AMR: antimicrobial resistance
AMS: antimicrobial stewardship

## 1 INTRODUCTION

Antimicrobial resistance (AMR) is considered to be the main cause of morbidity and mortality from treatable infections due to the overuse, or misuse of antimicrobials [1, 2]. AMR has an impact on the healthcare system by increasing the morbidity rate and the duration of hospitalization [1, 3]. AMR could be the biggest challenge facing healthcare in this century [4]. AMR has direct consequences including extended hospitalization, increased treatment expenses, and duration. Longer hospital stays need the use of additional antibiotics, which can result in side effects [5]. Thus, four factors are identified to explain the future extent of AMR including microbial and pathogenic ecology; prescribing and dispensing practices; characteristics of the population; and health policy [6]. Within this context, the World Health Organization (WHO) introduced several initiatives and programs to reduce AMR by educating healthcare providers to ensure appropriate prescribing of antimicrobials for appropriate indications [7]. In Iraq, there were signs of antibiotic overuse by healthcare practitioners, as well as inadequate control programs, which resulted in the rapid spread of resistant bacteria and the inability to regulate AMR. The Iraqi Ministry of Health approved the creation of an AMR National Coordinating Committee in 2017, which included policymakers from the human health, animal health, and environmental sectors, as well as the private sector. Its goal is to establish a healthy nation with appropriate access to excellent medical services and effective treatments to avoid the emergence and spread of AMR among the Iraqi people [8]. Antimicrobial stewardship (AMS) is a strategy designed to support the rational use of antimicrobials [9]. AMS programs can enhance antibiotic use in terms of antibiotic selection, dose, duration, and route of administration, while also lowering costs and minimizing antimicrobial side effects [10]. The appropriate use of antimicrobials will improve patient's health outcomes, and reduce the development of AMR [7].

Researchers have encouraged healthcare practitioners to incorporate AMS into their practices. Pharmacists work to enhance health, promote patient safety, and protect the public from infections [11, 12]. A systemic review by Saha et al. claimed that focusing on training and rules, collaboration between prescribers and community pharmacists, and identifying the roles of community pharmacists in AMS are essential for promoting good pharmacy practice. This review also suggested looking into the methods for involving community pharmacists in AMS and developing a collaboration with prescribers to combat AMR in primary care [13]. A study by MacMillan et al. revealed that optimizing antimicrobial prescribing practices in the pediatric emergency department can be achieved through pharmacist‐led interventions. According to this study, pharmacist interventions improved patient care in pediatric emergency by promoting the appropriate use of antimicrobials [14]. Community pharmacists are well‐placed within the community, having the capacity to identify public health issues and influence the management of acute and chronic diseases and other medical conditions at the community level [15]. Previous studies showed that there was a gap in community pharmacists' perception and practice regarding AMS [16, 17]. This includes the lack of involvement in awareness campaigns on AMS and the poor collaboration between pharmacists and other healthcare professionals regarding the use of antimicrobials [16]. However, few studies have been conducted elsewhere to investigate the role and perception of community pharmacists regarding AMS [18], but there is a lack of data in Iraq. Because of the potential role of community pharmacists in the development and implementation of AMS programs, it is very important to improve the role of pharmacists and their perception regarding AMS in community pharmacy.

## 2 METHODS

### 2.1 Research design

A cross‐sectional study was conducted from March to September 2021 to assess the role of pharmacists and their perception toward AMS in community pharmacies in Baghdad, Iraq.

### 2.2 Sampling method and sample size

A convenience sampling method was used to generate the sample size of the participants in this study. All community pharmacists from both genders in Baghdad City were eligible to this study. Community pharmacists who are working in community pharmacy as owner or employee were selected to participate in the survey. However, hospital pharmacists, patients, and policymakers were excluded from the study. The sample size was calculated by using the Raosoft sample‐size calculator, in which the population was taken as 6220 registered pharmacists in Baghdad, out of 11,347 registered pharmacists in Iraq [19].

(1) $$n=N\frac{x}{(N-1)E^{2}+x},$$

where *N* is the population size, *x* is the CI, and *E* is the margin of error. Based on the above formula, the sample size was calculated to be 362 participants when the power is kept at 80% and response distribution of 50%, with 95% confidence interval (CI) and 5% margin of error. An additional 20% was added to the sample size to account for the possibility of nonresponse or missing data to be in total of 435 participants.

### 2.3 Questionnaire development

The questionnaire was developed based on previous studies regarding the perception and practice of community pharmacists about AMS [16, 17]. The questionnaire was discussed via consultation with experts in the field of pharmacy practice by reviewing the relevant published studies relating to AMS. The content validity of the questionnaire was established by two lecturers who are subject matter experts. The questionnaire was pretested with five pharmacists to ensure the clarity of the questions and to include their suggestions in the concept of the study. A pilot study was carried out to confirm the validity and reliability of the items. The internal consistency of items was calculated by Cronbach's *α* coefficient. The value of Cronbach's *α* for eight items of perception was 0.736, and for nine items of the role of pharmacists was 0.909. The results of the pilot study were not included in the main study. The final version of the questionnaire included three major domains including the demographic characteristics of the participants, perception toward AMS, and the role of pharmacists in AMS.

### 2.4 Data collection

Data were collected from community pharmacists during their working hours in community pharmacies. The aim of the study was explained to all participants and their verbal consent was obtained before collecting data. A self‐administered questionnaire was used to collect the data from all the participants. The questionnaire was provided in English based on the participants' request to make the questions more understandable and easier for them to answer. The questionnaire was distributed to all participants to complete and return to the researcher at the same time. The questionnaire took approximately 20 min to be completed by the participants. Ethical approval was obtained from the Syndicate of Iraqi Pharmacists before commencement of the study.

### 2.5 Data analysis

The data was analyzed by the Statistical Package for Social Sciences (SPSS) program version 18.0 using appropriate descriptive and inferential statistics tests. Likert scale ranging from strongly agree to strongly disagree was used to describe the responses of specified statements of perception; and the following responses “always, often, sometimes, rarely, and never” were used to assess the role of pharmacists. The perception items were scored as “5 for strongly agree,” “4 for agree,” “3 for neutral,” “2 for disagree,” and “1 for strongly disagree”; while opposite scoring was only given for item 6. While, the items for the role of pharmacists were scored as “5 for always,” “4 for often,” “3 for sometimes,” “2 for rarely,” and "1 for never.” For analysis purposes, the total score of perception and pharmacists' role was divided into two categories “good and bad” based on the mean value of the total score. Nonparametric tests were employed to identify differences between the independent variables in the presence of skewness in the data. Mann–Whitney test was applied to find the difference between gender, position, and pharmacy type; while the Kruskal–Wallis test was computed to find the difference between age groups, qualifications, and experience years. Logistic regression was applied to predict the factors that were most likely to have an impact on pharmacists' role and perception regarding AMS. Statistical tests were considered significant based on 95% CI and *p* < 0.05.

## 3 RESULTS

### 3.1 Demographic characteristics of the participants

Out of 435 community pharmacies visited, 381 of them successfully participated in the survey, giving a response rate of 87.85%. From Table 1, the socio‐demographic characteristics of the participants showed that 73% of the participants were under the age of 31 years. More than half of the participants were male (55.9%). The majority of the participants (86.6%) had a bachelor's degree of pharmacy from any public or private university and 1.8% of them had completed their postgraduate studies (MSc in pharmacy), whereas 5.8% of them had a diploma in pharmacy (2 years program after high school that lead to a technical diploma) and 5.8% had a degree in different science fields. With regard to the position, 88.5% were pharmacists. Related to the experience years of the participants in community pharmacy, 31.8% of them had less than 3 years of experience, and 32.5% of them had 3–5 years of experience in community pharmacy. In addition, 56.7% of the participants were working in a medical complex pharmacy (medical complex consists of several private clinics).

**Table 1:** Socio‐demographic characteristics of the participants.

Variable		*N* (%)
Age group	≤30	278 (73.0)
	31–40	48 (12.6)
	>41	55 (14.4)
Gender	Male	213 (55.9)
	Female	168 (44.1)
Education	Diploma of pharmacy	22 (5.8)
	BSc of pharmacy	330 (86.6)
	Postgraduate (MSc or PhD)	7 (1.8)
	Others	22 (5.8)
Position	Pharmacist	337 (88.5)
	Assistant pharmacist (nonpharmacist)	44 (11.5)
Years of experience	<3	121 (31.8)
	3–5	124 (32.5)
	6–10	70 (18.4)
	>10	66 (17.3)
Type of pharmacy	Medical complex pharmacy	216 (56.7)
	Independent pharmacy	165 (43.3)

### 3.2 Perception toward antibiotic stewardship programs

From Table 2, most of the participants (85.6%) strongly agreed/agreed that “AMS programs reduce the problems of antibiotic resistance”; and 68% of them strongly agreed/agreed that AMS programs can improve patient care. In addition, 79.2% of the participants strongly agreed/agreed that AMS should be focused on the community pharmacy level; and 85.5% of them strongly agreed/agreed that community pharmacists required adequate training on antibiotics use. A high percent of participants (88.4%) strongly agreed/agreed that pharmacists have a responsibility to take a prominent role in AMS programs and infection‐control programs in the health system. Similarly, 88.4% of the participants strongly agreed/agreed that pharmacists should take an active role in AMS to reduce the impact of antibiotic resistance problems; and 91.4% of them strongly agreed/agreed that community pharmacists should attend educational training and programs to improve understanding of AMS. However, a high percent (84.8%) strongly disagreed/disagreed with the statement that only the prescribers need to understand AMS.

**Table 2:** Perception toward antimicrobial stewardship (AMS) programs.

Items	SA (%)	A (%)	N (%)	D (%)	SD (%)	Median (interquartile range)
1. AMS programs can improve patient care.	91 (23.9)	168 (44.1)	89 (23.4)	33 (8.7)	0 (0)	4 (3)
2. AMS should be focused on the community‐pharmacy level.	124 (32.5)	178 (46.7)	68 (17.8)	0 (0)	11 (2.9)	4 (4)
3. AMS programs reduce the problems of antibiotic resistance.	147 (38.6)	179 (47.0)	44 (11.5)	11 (2.9)	0 (0)	4 (4)
4. Adequate training should be provided to community pharmacists on antibiotics use.	159 (41.7)	167 (43.8)	44 (11.5)	0 (0)	11 (2.9)	4 (4)
5. Pharmacists have a responsibility to take a prominent role in antibiotic stewardship programs and infection‐control programs in the health system.	202 (53.0)	135 (35.4)	11 (2.9)	22 (5.8)	11 (2.9)	5 (4)
6. Healthcare professionals other than prescribers do not need to understand AMS.	12 (3.1)	0 (0)	46 (12.1)	201 (52.8)	122 (32.0)	4 (4–5)
7. Pharmacists should take an active role in AMS to reduce the impact of antibiotic resistance problem.	169 (44.4)	168 (44.1)	22 (5.8)	11 (2.9)	11 (2.9)	4 (4–5)
8. Community pharmacists should attend educational training and programs to improve understanding of AMS.	158 (41.5)	190 (49.9)	33 (8.7)	0 (0)	0 (0)	4 (4–5)

Abbreviations: A, agree; D, disagree; N, neutral; SA, strongly agree; SD, strongly disagree.

### 3.3 The role of pharmacists in antibiotic stewardship

From Table 3, almost two‐thirds of the participants (64.9%) reported always/often considering clinical and safety parameters like drug interactions, adverse drug reactions (ADRs), and allergies before dispensing the antibiotics. Similarly, around 62% of the participants always/often make efforts to prevent or reduce the transmission of infections within the community. More than half of the participants (55.9%) always/often participate in antimicrobial awareness movements to promote the rational use of antimicrobials. With regard to the dispensing of antimicrobials, 61.2% of the participants always/often communicate with prescribers when unsure about the appropriateness of an antibiotic prescription; and more than half (56.7%) of the participants always/often dispense antimicrobials on prescription with complete clinical information. Additionally, 52.8% of the community pharmacists educate patients on the use of antimicrobials and resistance‐related issues; and around 45.4% advise patients to continue the full course of antimicrobials. However, only 37.8% of the community pharmacists reported collaboration with other healthcare providers on antimicrobial use. It was also observed that only 43.5% of the community pharmacists evaluate antimicrobial prescriptions in accordance with the Iraqi national antimicrobial guidelines.

**Table 3:** The role of pharmacists in antimicrobial stewardship (AMS).

Items	Always	Often	Sometimes	Rarely	Never	Median (interquartile range)
1. I evaluate the antimicrobial prescription in accordance with the national antimicrobial guideline.	44 (11.5)	122 (32.0)	112 (29.4)	44 (11.5)	59 (15.5)	3 (2)
2. I collaborate with other healthcare professionals for AMS and infection prevention.	67 (17.6)	27 (20.2)	123 (32.3)	66 (17.3)	48 (12.6)	3 (2)
3. I communicate with prescribers if I am unsure about the appropriateness of an antibiotic prescription.	88 (23.1)	145 (38.1)	45 (11.8)	57 (15.0)	46 (12.1)	4 (2)
4. I consider clinical and safety parameters like drug interaction, ADRs, and allergy before dispensing the antibiotic prescribed.	177 (46.5)	70 (18.4)	13 (3.4)	121 (31.8)	0 (0)	4 (2)
5. I take part in antimicrobial awareness movements to promote the rational use of antimicrobials.	112 (29.4)	101 (26.5)	78 (20.5)	90 (23.6)	0 (0)	4 (3)
6. I make efforts to prevent or reduce the transmission of infections within the community.	114 (29.9)	122 (32.0)	44 (11.5)	99 (26.0)	2 (0.5)	4 (2)
7. I advise the patient to continue the full course of antimicrobial.	138 (36.2)	35 (9.2)	142 (37.3)	55 (14.4)	11 (2.9)	4 (3)
8. I dispense antimicrobials on prescription with complete clinical information.	169 (44.4)	47 (12.3)	77 (20.2)	88 (23.1)	0 (0)	4 (3)
9. I educate patients on the use of antimicrobials and resistance‐related issues.	123 (32.3)	78 (20.5)	81 (21.3)	88 (23.1)	11 (2.9)	4 (2)

From Table 4, the total score of perception toward AMS the role of pharmacists in AMS were influenced by the demographic data of the participants. There were significant differences between the total score of perception and age group, qualification, position, and years of experience (*p* < 0.001). A higher score was significantly influenced by older age group, higher degree in pharmacy, position as a pharmacist, and 6–10 years of experience. With regard to the role of pharmacists in AMS, there were significant differences between the total score of the role of pharmacists and age group, gender, qualification, years of experience, and pharmacy type (*p* < 0.05). A higher score was significantly influenced by younger age group, male participants, higher degree in pharmacy, 3–5 years of experience, and medical complex pharmacy.

**Table 4:** Factors affecting perception and the role of pharmacists regarding antimicrobial stewardship (AMS).

Socio‐demographic	Perception score (mean ± SD)	Median	*p*‐value	Role of pharmacists score (mean ± SD)	Median	*p*‐value
Age group						
≤30	31.26 ± 4.19	32.0	<0.001*	35.55 ± 6.51	37.0	<0.001*
31–40	35.45 ± 3.87	35.5		30.65 ± 10.0	33.0	
>41	40.00 ± 0.00	40.0		18.00 ± 0.00	18.0	
Gender						
Male	33.10 ± 5.32	33.0	0.287	33.13 ± 8.75	35.0	0.022*
Female	33.52 ± 4.48	33.0		30.83 ± 9.72	33.0	
Qualification						
Diploma of pharmacy	32.50 ± 0.51	32.5	<0.001*	30.71 ± 8.61	30.0	0.001*
BSc of pharmacy	33.60 ± 5.18	33.0		32.03 ± 9.55	34.0	
Postgraduate (MSc or PhD)	34.85 ± 3.13	35.0		38.00 ± 3.07	38.0	
Others	29.00 ± 0.00	29.0		28.00 ± 0.00	28.0	
AMS						
Pharmacist	33.62 ± 5.15	33.0	<0.001**	33.00 ± 5.49	33.0	0.735
Assist. pharmacist (nonpharmacist)	30.75 ± 1.80	30.5		32.00 ± 9.53	30.5	
Experience years						
<3	31.63 ± 3.43	31.0	<0.001*	32.82 ± 7.33	33.0	<0.001*
3–5	30.74 ± 4.95	32.0		37.20 ± 4.05	38.0	
6–10	34.98 ± 3.92	34.0		30.97 ± 10.22	33.0	
>10	39.33 ± 1.50	40.0		22.50 ± 10.13	18.0	
Pharmacy type						
Medical complex pharmacy	33.41 ± 3.31	33.0	0.831	35.72 ± 7.53	38.0	<0.001**
Independent pharmacy	33.13 ± 6.54	32.0		27.40 ± 8.96	38.0	

*Note*: *Kruskal–Wallis test *p* < 0.05; **Mann–Whitney test *p* < 0.05.

From Table 5, logistic regression was applied to predict the factors influencing how AMS was perceived by the community pharmacists. Accordingly, older age groups were more likely to have a good perception toward antibiotic stewardship compared to younger age groups (*p* < 0.001). In addition, participants with a qualification of a postgraduate degree (MSc of pharmacy) were two times more likely to have a higher score of perception compared to participants with other qualifications (odds ratio (OR) = 2.15, 95% CI: 0.71–1.21, *p* < 0.001). Pharmacists who have 6–10 years were more likely to have good perception (OR = 2.62, 95% CI: 1.38–1.95, *p* < 0.001). However, the position of participants had no impact on the perception toward antibiotic stewardship (*p* > 0.05).

**Table 5:** Predicting factors that influenced the perception toward antimicrobial stewardship.

Variables		Odds ratio (95% CI)
Age group	≤30	1.00
	31–40	3.69 (1.76–2.76)
	≥41–50	5.64 (1.18–1.32)*
Qualification	Diploma of pharmacy	1.00
	BSc of pharmacy	1.41 (0.52–0.77)
	Postgraduate (MSc or PhD)	2.15 (0.71–1.21)*
	Others	1.01 (0.35–0.62)
Position	Pharmacist	1.00
	Assist. pharmacist (nonpharmacist)	0.95 (0.10–0.14)
Years of experience	<3	1.00
	3–5	1.08 (0.56–1.05)
	6–10	2.62 (1.38–1.95)*
	>10	2.27 (0.24–0.27)

*Note*: *Statistically significant *p* < 0.05.

From Table 6, the factors were examined in this model to identify the predictors that significantly contributed to the role of pharmacists in antibiotic stewardship. A higher score of the role of pharmacists reflected good pharmacy practice regarding antibiotic stewardship. The younger age group (24–30 years) was more likely to have a higher score for role of pharmacists compared to older age groups (*p* < 0.001). However, female pharmacists were less likely to have higher scores comparing to male pharmacists (OR = 0.21, 95% CI: 0.20–0.33, *p* < 0.001). In addition, participants with a higher degree in pharmacy whether “Masters or PhD” were more likely to have a higher score of the role of pharmacists compared to participants with other qualifications (OR = 2.50, 95% CI: 1.01–6.15, *p* < 0.001). Pharmacists who have 3–5 years were more likely to have a higher score of the role of pharmacists regarding antibiotic stewardship (OR = 7.27, 95% CI: 1.99–2.69, *p* < 0.001). On the other hand, pharmacists who work in independent pharmacies were less likely to have higher scores comparing to pharmacists who work in medical complex pharmacies (OR = 0.06, 95% CI: 0.03–0.13, *p* < 0.001).

**Table 6:** Predicting factors that influenced the role of pharmacists in antimicrobial stewardship.

Variables		Odds ratio (95% CI)
Age group	≤30	1.00*
	31–40	0.41 (0.22–0.76)
	≥41–50	0.29 (0.19–0.32)
Gender	Male	1.00
	Female	0.21 (0.20–0.33)*
Qualification	Diploma of pharmacy	1.00
	BSc of pharmacy	1.79 (0.72–4.47)
	Postgraduate (MSc or PhD)	2.50 (1.01–6.15)*
	Others	1.32 (0.50–3.46)
Years of experience	<3	1.00
	3–5	7.27 (1.99–2.69)*
	6–10	1.37 (0.67–2.79)
	>10	0.10 (0.04–0.27)
Pharmacy type	Medical complex pharmacy	1.00
	Independent pharmacy	0.06 (0.03–0.13)*

*Note*: *Statistically significant *p* < 0.05.

## 4 DISCUSSION

Community pharmacists are expected to dispense antimicrobials appropriately and take a positive role in AMS. This study showed that the majority of the pharmacists agreed that AMS programs reduce the problems of antibiotic resistance and improve patient care. In Malaysia, similar findings were reported by Khan et al. in which more than 90% of pharmacists agreed with the importance of AMS for improving patient care [18]. AMS is important for promoting the appropriate use of antibiotics, enhancing patient outcomes, and reducing antibiotic resistance [17, 20]. In the current study, pharmacists also agreed that they have a major role in AMS programs and infection‐control programs in the health system; which was similar to what was found in previous studies conducted elsewhere [17, 18]. According to an earlier study assessed pharmacists' role in intravenous to oral antimicrobial conversion programs showed excellent economic outcomes as well as a positive influence on patient health outcomes by reducing the duration of hospitalization and intravenous antibiotic therapy [21]. Other studies conducted in Malaysia and Pakistan showed that community pharmacists agreed with the importance of AMS to improve patient care [16, 18]. However, another study in Saudi Arabia indicated that the lack of internal policies and procedures and inadequate training were the biggest obstacles for AMS [22].

This study reported that most of the pharmacists agreed that AMS should be focused on the community‐pharmacy level, and they also agreed that adequate training on antibiotics use and attending programs to improve understanding of AMS are needed. Previous studies revealed that pharmacists want to be more involved in AMS [17, 18, 22]. This may be because community pharmacists can play a key role in encouraging the appropriate use of antibiotics [17, 18, 23], as well as creating strong lines of professional communication with prescribers for AMS programs [17, 24]. In the current study, a high percent of the pharmacists agreed that other healthcare professionals should also understand AMS. Pharmacists disagreed that physicians are the only professionals who need to understand AMS and they believed that pharmacists have an active role in improving antibiotic use [17, 25]. Antibiotic dispensing must be strictly regulated by community pharmacists to tackle the issue of antibiotic resistance.

It's also found that pharmacists from older age groups were more likely to have a good perception toward antibiotic stewardship compared to younger age groups. In addition, pharmacists with postgraduate degrees had a significantly higher score of perception compared to participants with other qualifications. Similarly, pharmacists with 6–10 years of experience had considerably good perception. These findings agreed with a study by Erku that the age group, qualifications, and years of experience were significantly associated with higher scores with respect to perception. This could clarify how pharmacy education, experience years, and knowledge can help pharmacists to understand AMS [17].

The findings of this study also showed that many pharmacists consider clinical and safety parameters like drug interactions, ADRs, and allergies before dispensing antibiotics. Previous studies showed that pharmacists have a positive role in improving clinical outcomes in many countries where AMS is implemented [26]. Their role was to reduce the inappropriate use of antibiotics, the risk of resistance and adverse events [27], the prevalence of resistant bacteria, and the cost of healthcare [26, 28]. Another study by Ahmed et al, found that AMS programs improved prescriber's adherence to the guidelines and recommendations. Additionally, it improved the appropriate use of antibiotics, prescription of the recommended dosage, frequency and duration of prophylaxis, and choice of administration route [29]. In this study, around half of the participants tend to advise the patient to continue the full course of antimicrobials; and to dispense antimicrobials on prescription with complete clinical information. In Pakistan, a study by Akbar et al. showed that the majority of community pharmacists have a positive role in educating patients about antibiotic use and resistance‐related issues, thus advising them to continue the antibiotic course [30]. This study also revealed that around 62% of pharmacists make efforts to prevent or reduce the transmission of infections within the community, which is similar to the findings found in other studies conducted [17, 25]. In the present study, more than half of the pharmacists participated in antimicrobial awareness movements to promote the rational use of antimicrobials. Similar results were found in previous studies [17, 25]. In Australia, a study by Cotta et al. showed that community pharmacists were more excited to take part in AMS movements in healthcare settings [31].

With regard to the dispensing of antimicrobials, around 60% of the pharmacists in this study always/often communicate with prescribers when unsure about the appropriateness of an antibiotic prescription. A study by Rehman et al. reported similar findings that pharmacists collaborate with physicians to ensure the choice of antibiotic is appropriate [25]. This is in contrast to a study in Pakistan that fewer pharmacists tend to collaborate with prescriber to check the appropriateness of the prescribed antibiotic [16]. The current study also showed that pharmacists were less likely evaluate the antimicrobial prescription in accordance with the national antimicrobial guideline and few of them collaborate with other healthcare professionals for AMS and infection control. Previous studies also showed that pharmacists are less likely to collaborate with other healthcare professionals about AMS and infection control [16, 18, 25]. This may explain that why some pharmacists are still in doubt about the potential benefits of collaborative practice with other healthcare professionals for AMS [16].

## 5 STUDY LIMITATIONS

This study was limited to the role of pharmacists and their perception relating to stewardship of prescribed antibiotics. Nonprescription sales of antibiotics were not assessed due to the lack of strict rules and regulations in the country, which allow antibiotics to be obtained on patient's request. The role of pharmacists was assessed based on a self‐administered questionnaire; so, participants' responses might be influenced by their tendency to select the most socially acceptable responses and may not reflect the actual role of pharmacists as antimicrobial stewards in the community pharmacy. In addition, this study was not focused on the impact of national antibiotic stewardship in the country. So, pharmacists' responses about AMS might be influenced by other contributing resources such as private training programs, the internet or social media. This study predicted factors that influence the role and perception of pharmacists; other confounding factors may also have an impact; however, they were not included in this study.

## 6 CONCLUSION

Since 2017, AMS is a component of the national plan to reduce AMR in Iraq. The implementation of AMS strategies in community pharmacy is obviously poor. In this study, community pharmacists showed good perception toward AMS, but their engagement with AMS was still poor. More efforts and attention from healthcare authorities should be considered to optimize the use of antibiotics in the community. With the allowance for nonprescription antibiotics, it highlights the need for community pharmacists to become active antimicrobial stewards. Therefore, AMS should be addressed in pharmacy education to ensure the appropriate use of antibiotics, enhance patient safety, and reduce costs to the community and patients. Pharmacists and other health care professionals should collaborate together in multidisciplinary teams in AMS programs to reduce resistance and inappropriate use of antimicrobials. The national AMS should be implemented to monitor the over‐prescribing and dispensing of antibiotics in community pharmacy in Iraq. Further surveys should be created to examine issues at different levels including individual practice, system, and policy to obtain an in‐depth qualitative understanding of the barriers that community pharmacists may face when conducting AMS.

## AUTHOR CONTRIBUTIONS

Akram Alkadhimi analyzed the data and prepared the first draft of the manuscript. Omar T. Dawood participated in the conception and design of the study, and constructively revised the manuscript. Akram Alkadhimi participated in data collection and organization. Amer H. Khan participated in and supervised the study throughout. All the authors commented on previous versions of the manuscript and approved the final version.

## CONFLICT OF INTEREST STATEMENT

The authors declare no conflict of interest.

## ETHICS STATEMENT

The study protocol was approved by the Ethics Committee of the Syndicate of Iraqi Pharmacists (7304/9/2019), and it was compliant with the Helsinki Declaration of 1975, as revised in 2008.

## INFORMED CONSENT

Not applicable.

## Data Availability

The data that support the findings of this study are available on request from the corresponding author. The data are not publicly available due to privacy or ethical restrictions.
